# Supplementary material for: Synthesis of Berberine and Canagliflozin Chimera and Investigation into New Antibacterial Activity and Mechanisms
Source: Molecules. 2022 May 5;27(9):2948. doi: 10.3390/molecules27092948 (PMC9100802; doi:10.3390/molecules27092948)
Supplement: Supplementary file 1 [file molecules-27-02948-s001.zip › molecules-1667831-supplementary.pdf]

Supporting Information For

Synthesis of Berberine and Canagliflozin chimera and investigation into new antibacterial activity and mechanisms

Wenhui Hao [#1], Shiying Che [#2,3,4], Jinsheng Li [2,3,4], Jingyi Luo [1,5], Wanqiu Zhang [1,5], Yang Chen [1,5], Zijian Zhao [*2,3,4], Hao Wei [*3] and Weidong Xie [*1,5]

1 State Key Laboratory of Chemical Oncogenomics, Shenzhen International Graduate School, Tsinghua Uni-versity, Shenzhen, Guangdong 518055; China

2 School of Chemistry and Materials Science, Huaihua University, Huaihua, Hunan 418000, China

3 College of Pharmacy, Shaanxi University of Chinese Medicine, Xi'an-Xianyang New Ecomic Zone, Xianyang, Shaanxi 712046, China

4 Key Laboratory of Research and Utilization of Ethnomedicinal Plant Resources of Hunan Province, Huai-hua University, Hunan, Huaihua 418000, China

5 Shenzhen Key Lab of Health Science and Technology, Institute of Biopharmaceutical and Health Engi-neering, Shenzhen International Graduate School, Tsinghua University, Shenzhen, Guangdong 518055, China

* Correspondence: E-mail: zjzhao72@163.com (Z.Z.), weihao217@163.com (H.W.), xiewd@sz.tsinghua.edu.cn (W.X.)

\# These authors contributed equally to this work

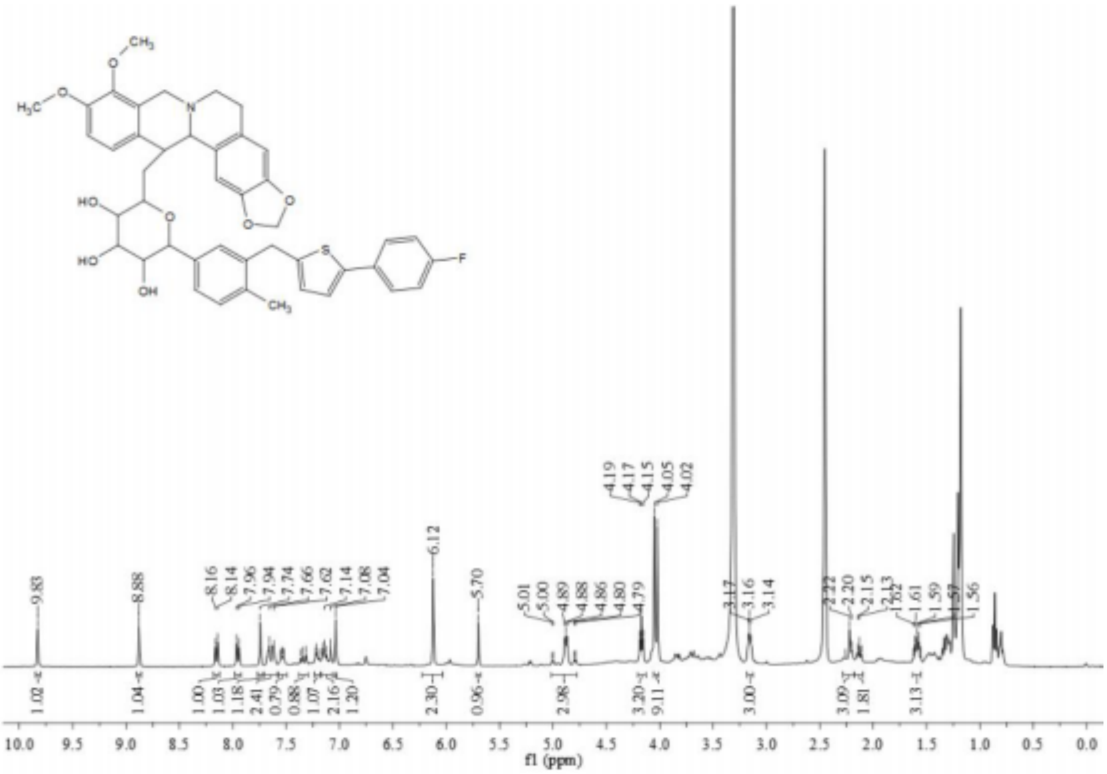

Figure S1 ^{1}H NMR spectra of compound BC

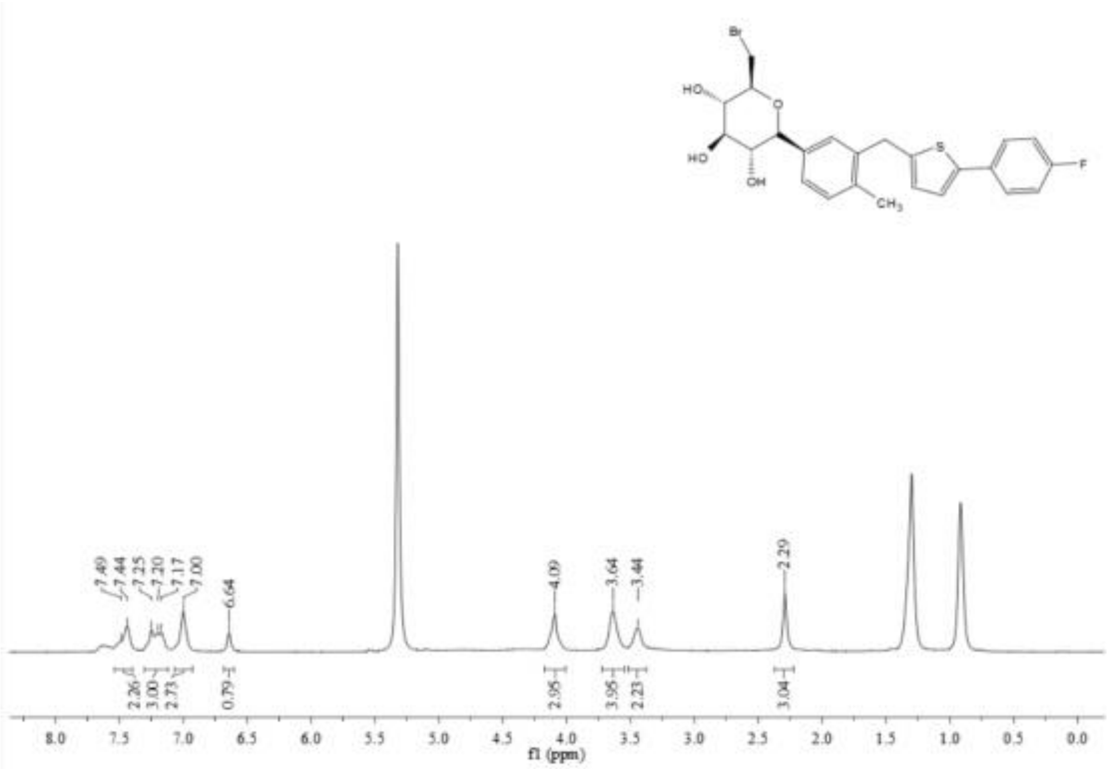

Figure S2 ^{1}H NMR spectra of compound CAN bromide

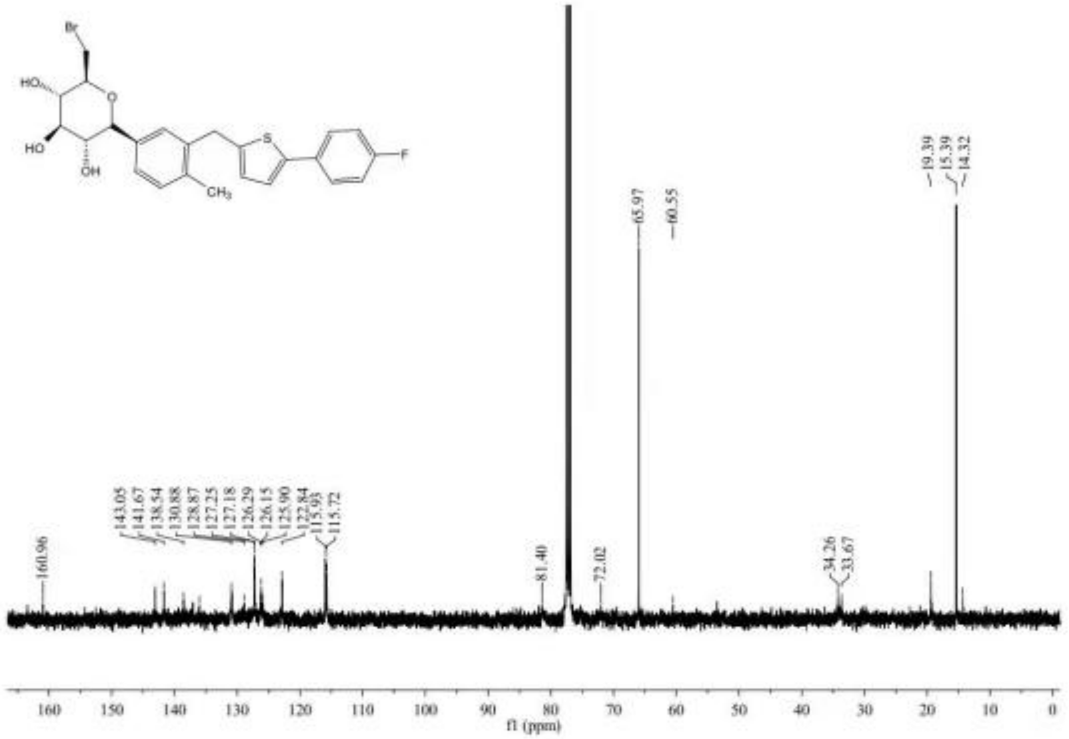

Figure S3 ^{13}C NMR spectra of compound CAN bromide

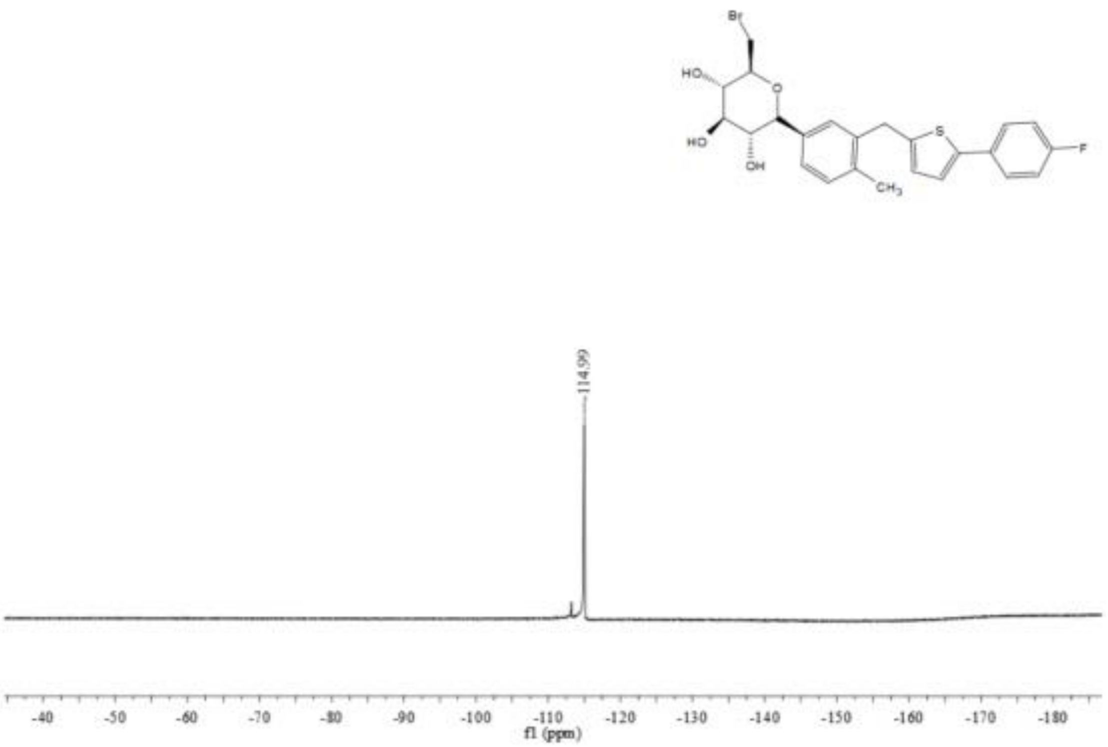

Figure S4 ^{19}F NMR spectra of compound CAN bromide

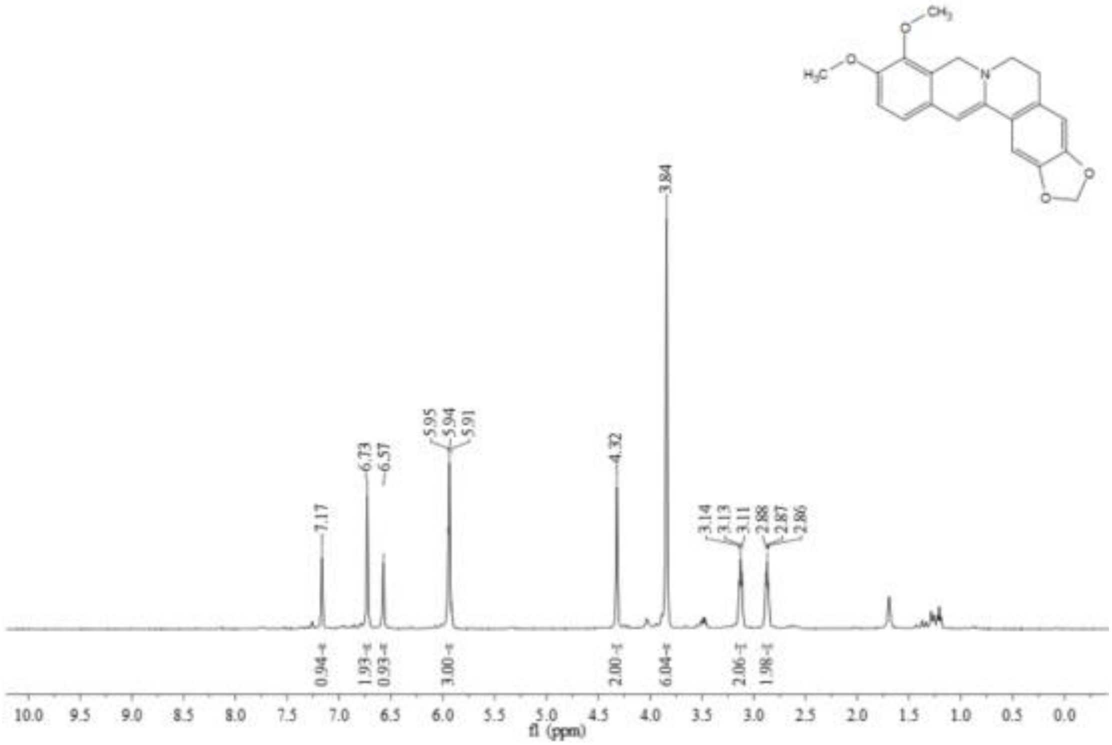

Figure S5 ^{1}H NMR spectra of compound BC

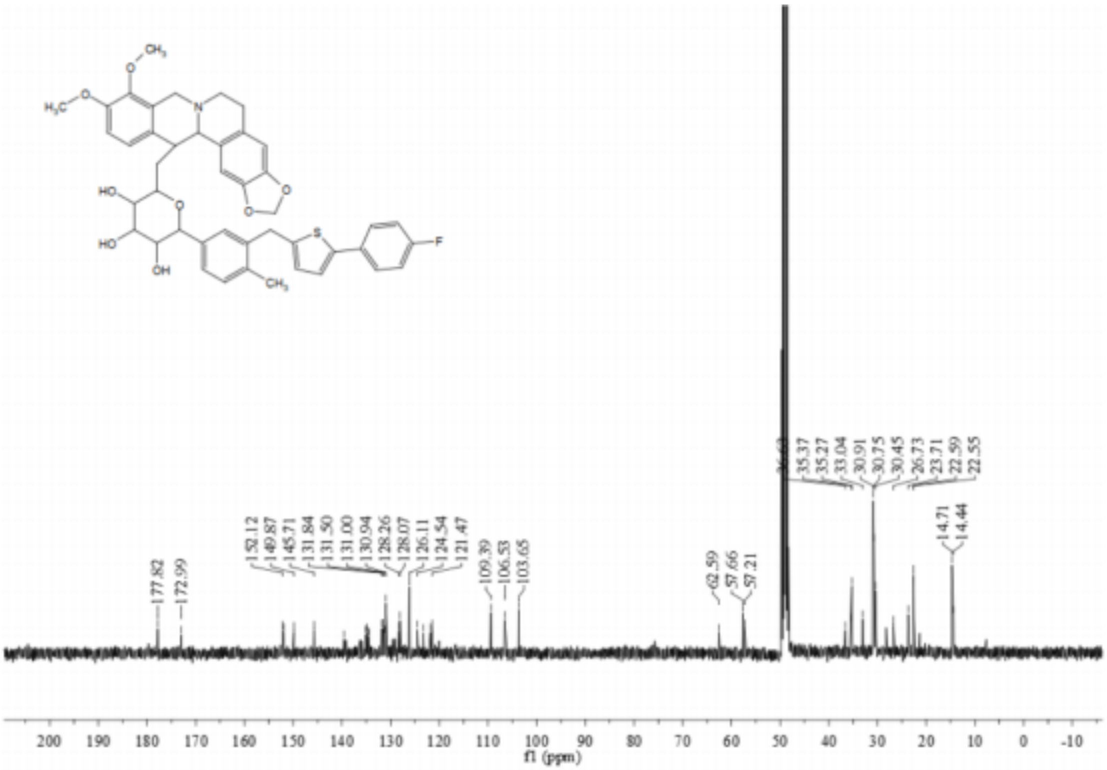

Figure S6 ^{13}C NMR spectra of compound BC

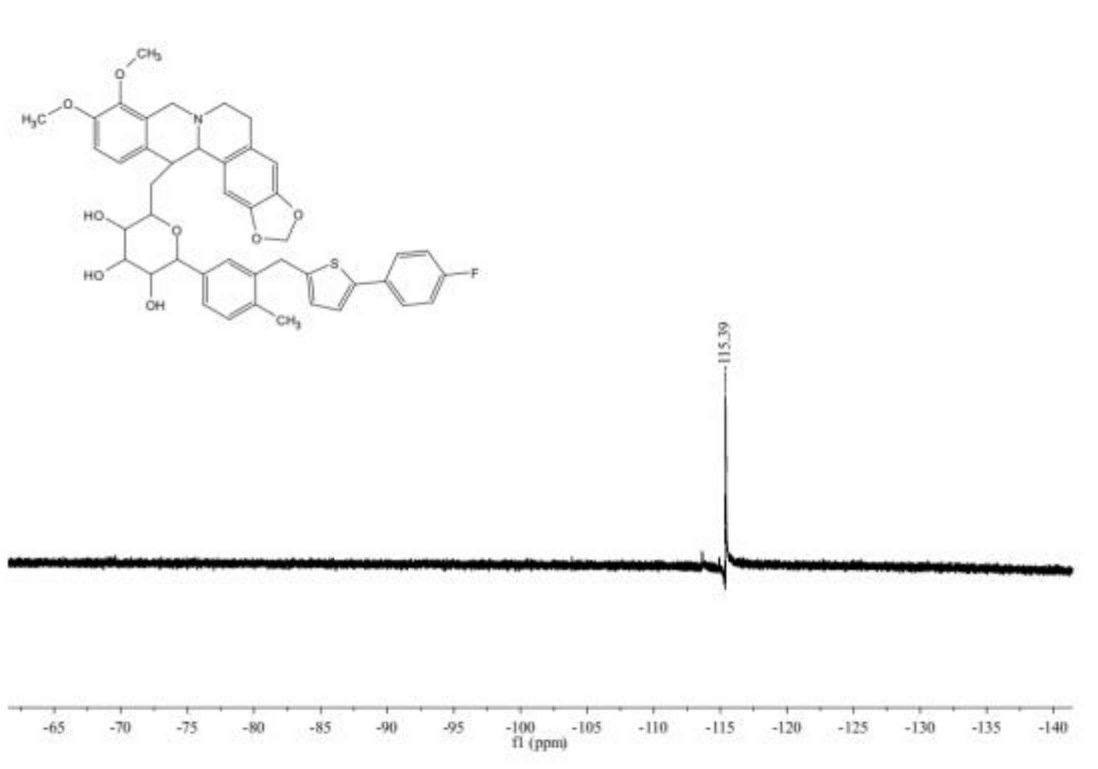

Figure S7 ^{19}F NMR spectra of compound BC

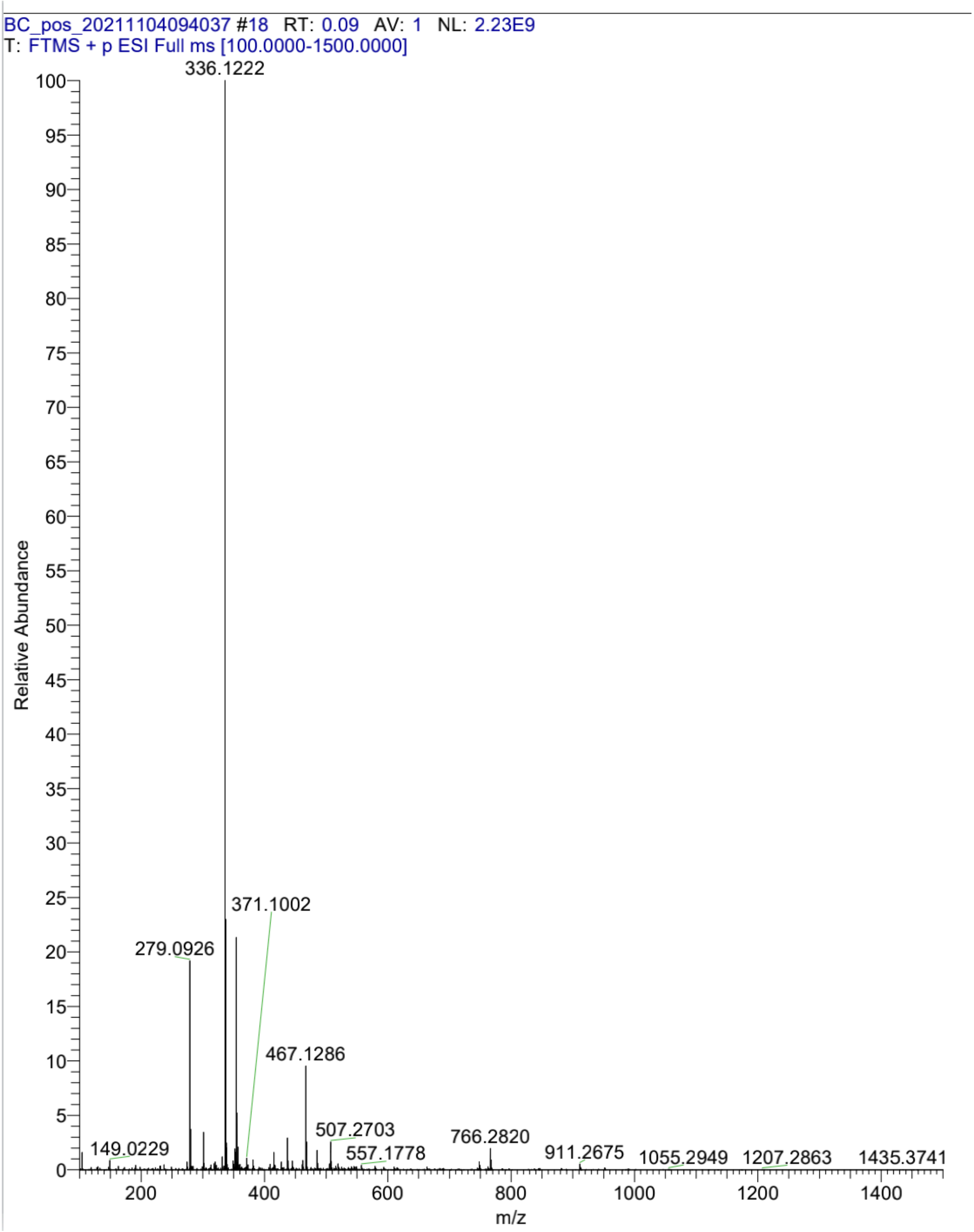

Figure S8 H RMS (FT)spectra of compound BC

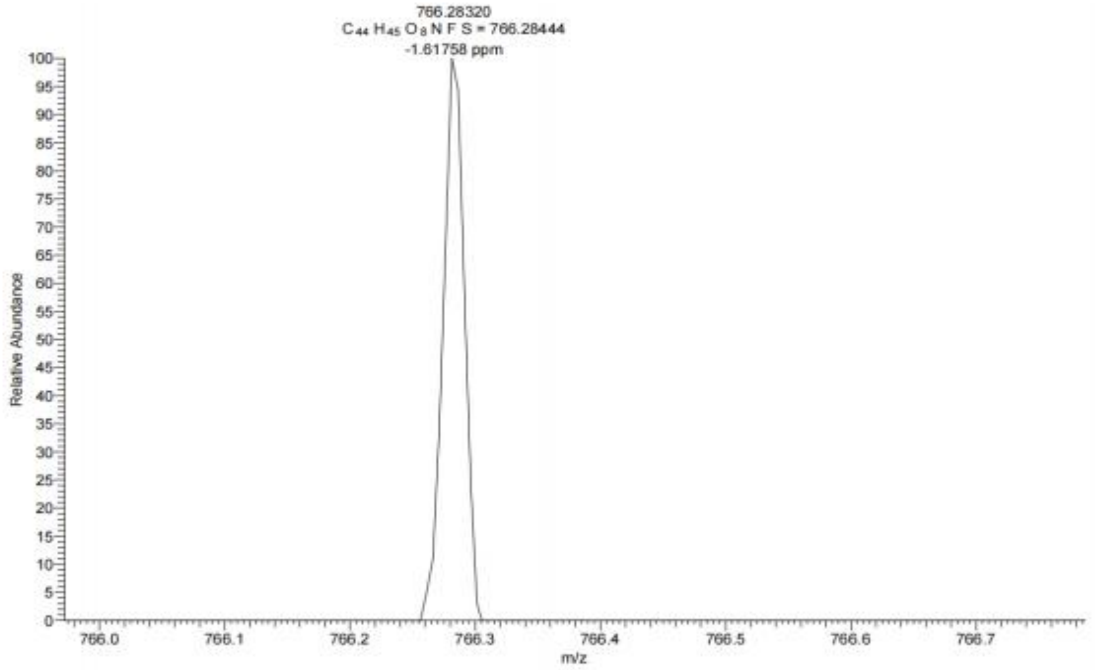

Figure S9 H RMS (ESI) spectra of compound BC

<色谱图> (Chromatogram)

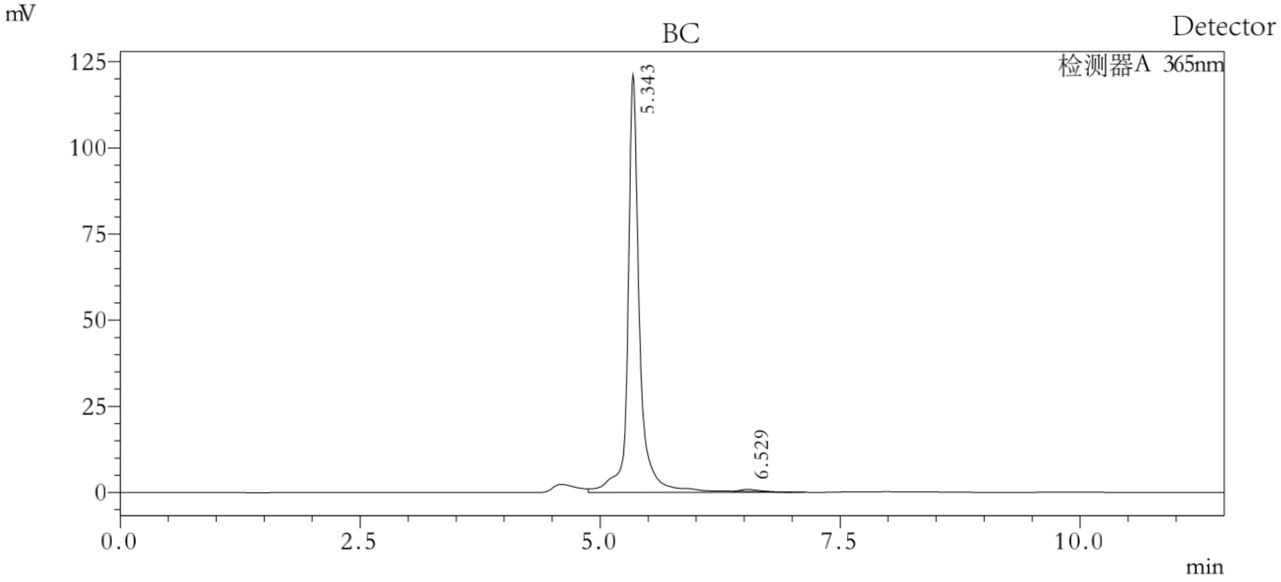

<峰表>

检测器A 365nm

峰号	保留时间	面积	高度	浓度	浓度单位	标记	化合物名
1	5.343	1031452	121149	99.175		S	
2	6.529	8585	614	0.825		TV	
总计		1040037	121763				
Peaks	Retention time	Area	Height	Concentration			

Figure S10 HPLC of BC at 365 nm

<色谱图> (Chromatogram)

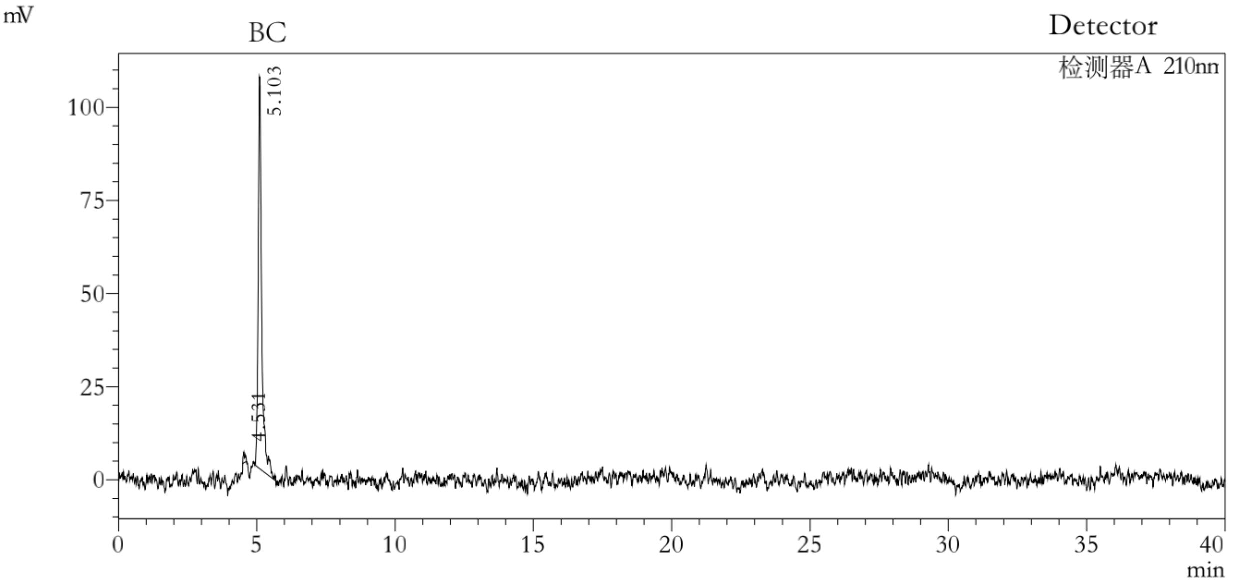

<峰表>

检测器A 210nm

峰号	保留时间	面积	高度	浓度	浓度单位	标记	化合物名
1	4.531	13337	2913	1.423		M	
2	5.103	923563	105191	98.577		M	
总计		936899	108104				

Peaks Retention time Area Height Concentration

Figure S11 HPLC of BC at 210 nm
